# HMGA1: A Master Regulator of Tumor Progression in Triple-Negative Breast Cancer Cells

**DOI:** 10.1371/journal.pone.0063419

**Published:** 2013-05-02

**Authors:** Sandeep N. Shah, Leslie Cope, Weijie Poh, Amy Belton, Sujayita Roy, C. Conover Talbot, Saraswati Sukumar, David L. Huso, Linda M. S. Resar

**Affiliations:** 1 Hematology Division, Department of Medicine, Johns Hopkins University School of Medicine, Baltimore, Maryland, United States of America; 2 Department of Oncology, Johns Hopkins University School of Medicine, Baltimore, Maryland, United States of America; 3 Department of Biostatistics, Johns Hopkins University School of Medicine, Baltimore, Maryland, United States of America; 4 Pathobiology Graduate Program, Johns Hopkins University School of Medicine, Baltimore, Maryland, United States of America; 5 Institute for Basic Biomedical Sciences, Johns Hopkins University School of Medicine, Baltimore, Maryland, United States of America; 6 Department of Pathology, Johns Hopkins University School of Medicine, Baltimore, Maryland, United States of America; 7 Department of Comparative Molecular & Pathobiology, Johns Hopkins University School of Medicine, Baltimore, Maryland, United States of America; 8 Department of Pediatrics, Johns Hopkins University School of Medicine, Baltimore, Maryland, United States of America; 9 Institute for Cellular Engineering, Johns Hopkins University School of Medicine, Baltimore, Maryland, United States of America; Virginia Commonwealth University, United States of America

## Abstract

Emerging evidence suggests that tumor cells metastasize by co-opting stem cell transcriptional networks, although the molecular underpinnings of this process are poorly understood. Here, we show for the first time that the *high mobility group A1* (*HMGA1*) gene drives metastatic progression in triple negative breast cancer cells (MDA-MB-231, Hs578T) by reprogramming cancer cells to a stem-like state. Silencing *HMGA1* expression in invasive, aggressive breast cancer cells dramatically halts cell growth and results in striking morphologic changes from mesenchymal-like, spindle-shaped cells to cuboidal, epithelial-like cells. Mesenchymal genes (*Vimentin, Snail*) are repressed, while *E-cadherin* is induced in the knock-down cells. Silencing *HMGA1* also blocks oncogenic properties, including proliferation, migration, invasion, and orthotopic tumorigenesis. Metastatic progression following mammary implantation is almost completely abrogated in the *HMGA1* knock-down cells. Moreover, silencing *HMGA1* inhibits the stem cell property of three-dimensional mammosphere formation, including primary, secondary, and tertiary spheres. In addition, knock-down of *HMGA1* depletes cancer initiator/cancer stem cells and prevents tumorigenesis at limiting dilutions. We also discovered an HMGA1 signature in triple negative breast cancer cells that is highly enriched in embryonic stem cells. Together, these findings indicate that HMGA1 is a master regulator of tumor progression in breast cancer by reprogramming cancer cells through stem cell transcriptional networks. Future studies are needed to determine how to target HMGA1 in therapy.

## Introduction

Despite advances in our ability to detect and treat breast cancer, it remains a leading cause of death in women with cancer, and the incidence is rising [1]. Approximately 15–20% of all cases are classified as triple negative breast cancer, a subtype that is frequently associated with rapid progression and poor outcomes [1]–[2]. Triple negative breast cancer refers to the lack of detectable markers for the estrogen receptor (ER), progesterone receptor (PR), and *Her2/neu* amplification. These tumors do not respond to our most effective and least toxic therapies, including hormonal therapy (tamoxifen) or herceptin. Thus, further studies are needed to elucidate the molecular pathways that lead to tumor progression in triple negative breast cancer and could be targeted with novel therapies.

Recent studies identified the *high mobility group A1* (*HMGA1*) oncogene as a key factor enriched in embryonic stem cells, adult stem cells, and refractory or high-grade/poorly differentiated tumors [3]–[32]. The *HMGA1* gene encodes the HMGA1a and HMGA1b chromatin remodeling proteins, which result from alternatively spliced messenger RNA [4], [14], [22], [27]. These low molecular weight (thus high mobility group) protein isoforms bind to the minor groove of chromatin at AT-rich regions. HMGA1 proteins modulate gene expression by altering chromatin structure and orchestrating the assembly of transcription factor complexes to enhanceosomes within enhancer or promoter regions throughout the genome. These proteins are highly expressed during embryogenesis with low or absent levels in adult tissues. *HMGA1* is overexpressed in all aggressive cancers studied to date, and high levels portend a poor prognosis in diverse tumors [3]–[31]. In fact, HMGA1 proteins are the most abundant nonhistone chromatin binding proteins found in cancer cells. A recent landmark paper demonstrated that *HMGA1* is essential for the cellular reprogramming of somatic cells to induced pluripotent stem cells by the four Yamanaka factors (Oct4, Sox2, Klf4, cMyc) [32]. HMGA1 induces expression of key stem cell transcriptional networks in normal embryonic stem cells and during cellular reprogramming. Together, these findings suggest that HMGA1 could function in tumor progression by reprogramming differentiated cells into poorly differentiated, stem-like cancer cells.

Here, we discovered that HMGA1 is a central factor in reprogramming poorly differentiated, triple-negative breast cancer cells. Our findings further implicate HMGA1 as a master regulator in tumor progression and suggest that targeting HMGA1 pathways could be effective in poorly differentiated, metastatic tumors.

## Results

### Silencing *HMGA1* halts cell proliferation and reprograms invasive, mesenchymal-like cells

**Figure 1:**
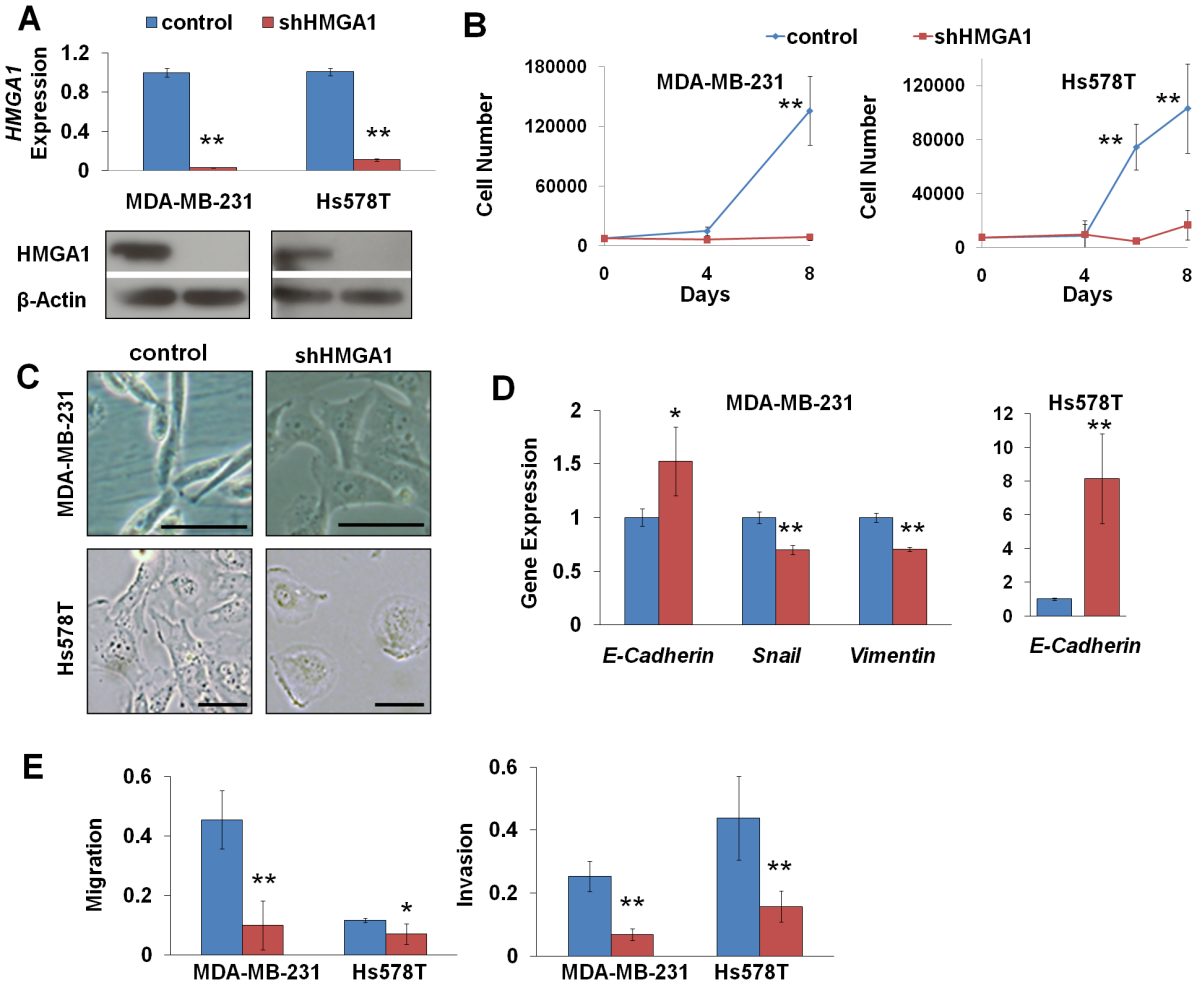
Silencing *HMGA1* expression halts cell growth and induces dramatic changes in cell morphology and gene expression. **A**) Lentiviral-mediated delivery of shRNA to *HMGA1* (denoted shHMGA1) results in a marked decrease in *HMGA1* mRNA and protein in triple negative breast cancer cell lines (MDA-MB-231, Hs578T). **B**) Proliferation is disrupted in cancer cell lines following silencing of *HMGA1*. **C**) Mesenchymal, fibroblast-like cancer cells undergo dramatic morphologic changes within 4 days after treatment with shHMGA1. Striking changes were observed in MDA-MB-231 (top panels) and Hs578T cells (bottom panels). Bar: 50 µm. **D**) Alterations in EMT genes with silencing of *HMGA1*. **E**) Migration and invasion is decreased with silencing of *HMGA1*. *P<0.05; **P<0.01.

To define the role of *HMGA1* in oncogenic properties and tumor progression, we silenced *HMGA1* expression using lentiviral-mediated delivery of short hairpin RNA (shRNA) [32] in cell lines derived from aggressive, triple negative breast cancers (MDA-MB-231, Hs578T; Fig. 1A). Control cells were transduced with a control lentiviral vector [10], [32]. We discovered that cell proliferation was rapidly halted in both cell lines (Fig. 1B) within the first 4 days. Surprisingly, there was a dramatic change in cell morphology whereby the spindle-shaped, fibroblast-like cells became cuboidal and epithelial-like in appearance (Fig. 1C). Because these morphologic changes are consistent with a mesenchymal-epithelial transition, we investigated the expression of genes involved in a mesenchymal-epithelial transition [10], [33]. In MDA-MB-231 cells, we found that silencing *HMGA1* led to a significant repression in the mesenchymal genes, *Snail* and *Vimentin*, while there was an increase in the gene expressing the epithelial marker, E-Cadherin (Fig. 1D). Similarly, in Hs578T cells, *E-Cadherin* was induced when *HMGA1* was silenced. We also assessed tumor progression properties, including invasion and migration. In both cell lines, there was a marked reduction in migration and invasion in cells with silencing of *HMGA1* (Fig. 1E). Together, these findings indicate that silencing *HMGA1* results in a profound decrease in proliferation, migration, and invasion, as well as morphologic and gene expression changes consistent with a mesenchymal-epithelial transition.

### Silencing *HMGA1* interferes with orthotopic tumorigenicity and metastatic progression

**Figure 2:**
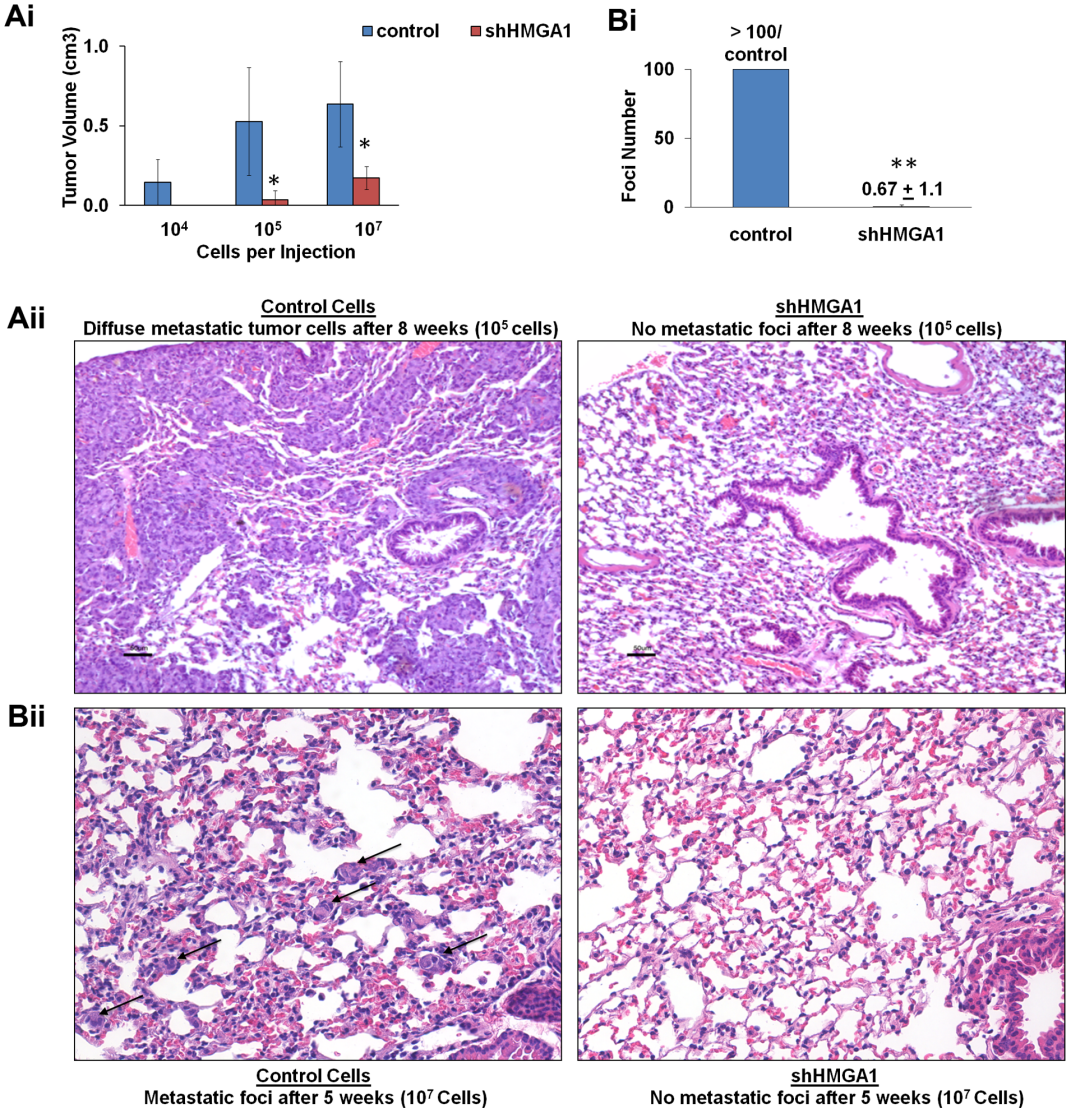
Silencing *HMGA1* interferes with orthotopic tumorigenicity and metastatic progression. **A)** Silencing *HMGA1* impairs orthotopic tumorigenicity. Tumor volumes±standard deviations are shown. No tumors formed from shHMGA1 cells when 10^4^ cells were implanted. (For injections with 10^4^ cells, n = 3 for control or shHMGA1 cells; for injections with 10^5^ cells, n = 5 for control and n = 8 for shHMGA1 cells; and for injections with 10^7^ cells, n = 3 for control and shHMGA1 cells. **B**) Metastatic progression is almost completely abrogated in cells that do not express *HMGA1.* This graph shows the number metastatic foci to the lung 5 weeks following implantation of MDA-MB-231 cells (10^7^) into mammary fat pads following treatment with control shRNA or shHMGA1. **C**) The top photographs show the lungs 8 weeks following implantation into mammary fat pads. There are coalescing sheets of metastatic tumor cells in the lungs of mice injected with control cells (left) as compared to mice injected with shHMGA1 cells (right). Due to the widespread tumor cells, individual foci could not be counted. Bar: 50 µm. **D**) The bottom panels show multiple, discreet foci in the lungs 5 weeks following implantation of control cells into mammary fat pads (left) as compared to mice injected with shHMGA1 cells (right). *P<0.05; **P<0.0001.

Next, we assessed the role of HMGA1 on tumorigenesis using *in vivo* models of triple negative breast cancer. First, we assessed tumor growth following mammary fat pad implantation. We found that silencing *HMGA1* in the aggressive MDA-MB-231 cells leads to a dramatic decrease in tumor growth following mammary fat pad implantation (Fig. 2Ai). Specifically, cells (10^5^) transduced with control virus reached a volume of 0.53 cm^3^±0.34 at 8 weeks following mammary fat pad implantation. In contrast, the tumors from cells transduced with *HMGA1* shRNA (shHMGA1) were significantly smaller at 8 weeks following implantation (0.037 cm^3^±0.058; p = 0.016). Because there was a dramatic effect on primary tumorigenesis, we also sought to determine if silencing *HMGA1* interferes with metastatic progression. We therefore evaluated the lungs histopathologically for tumor foci after necropsy. Strikingly, we discovered almost no metastatic lesions to the lungs in the mice implanted with the shHMGA1 cells as compared to the mice implanted with control cells in which there were extensive, coalescing sheets of metastatic tumor cells throughout the lungs following mammary implantation with 10^5^ cells (Fig. 2Aii). We also assessed metastatic progression following mammary fat pad implantation with a greater number of cells (10^7^) from a repeat transduction experiment, and sacrificed the mice after 5 weeks. With the higher number of cells, tumors formed from all injections (3/3 in controls and 3/3 in shHMGA1 cells (Fig. 2Bi). Although tumors were slightly smaller from the shHMGA1 cells, the difference was not significant (0.64±0.27 in controls versus 0.17±0.072 in shHMGA1 cells, p = 0.08). Despite the similar tumor volumes, we observed a dramatic decrease (>100-fold) in metastatic foci in the shHMGA1 cells as compared to controls (0.67±1.15 versus >100 in all controls; p = 0.00004; Fig. 2Bi & 2Bii). We also assessed lung foci following tail vein injection of control or shHMGA1 cells (10^6^) after 3 weeks. Only one lung focus was observed after injection of the shHMGA1 cells, while there were numerous foci in the control cells (0.25±0.5 versus 99.3±15.0; p = 0.007; Fig. S1).

### Silencing *HMGA1* blocks mammosphere formation and depletes tumor-initiator cells

**Figure 3:**
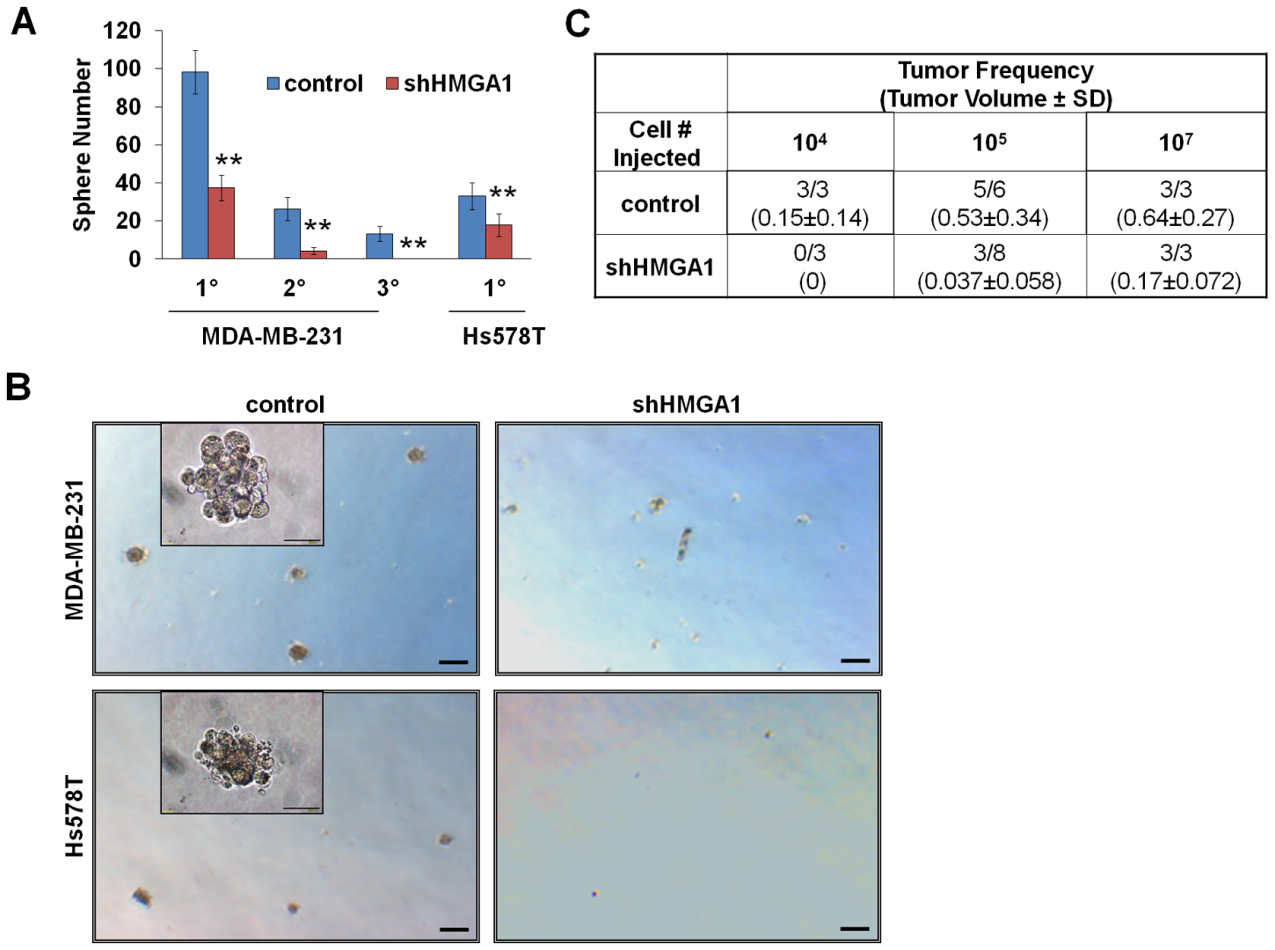
Silencing *HMGA1* blocks mammosphere formation and depletes tumor-initiator cells. **A**) Silencing *HMGA1* blocks mammosphere formation in MDA-MB-231 cells (1°, 2°, 3°) and Hs578T cells (1°). **B**) Photographs of mammospheres following treatment of breast cancer cells with control or shHMGA1. Silencing *HMGA1* significantly inhibits mammosphere formation in MDA-MB-231 and Hs578T cells. Bars: 200 µm (large panels) and 50 µm (insets). **C**) Tumor numbers at limiting dilutions show that silencing *HMGA1* depletes the tumor initiator/cancer stem cells in MDA-MB-231 cells. Note that no tumors formed following injection of 10^4^ cells treated with shHMGA1, while tumors formed in all cases when control cells were injected. Both tumor frequency and tumor volumes (± standard deviations) are shown. *P<0.05; **P<0.01.

Because silencing *HMGA1* has profound effects on oncogenic properties *in vitro,* primary tumorigenesis and metastatic progression *in vivo*, and expression of genes involved in epithelial-mesenchymal transition, we sought to determine its role in cancer stem cell characteristics. To this end, we explored the epithelial stem cell property of mammosphere formation [34] in the control and shHMGA1-treated cells (Fig. 3A–B). We found that growth of primary, secondary, and tertiary mammospheres was significantly impaired in the MDA-MB-231 cells with silencing of *HMGA1*. Similarly, we observed that there was a significant decrease in primary mammosphere formation in the Hs578T cells treated with shHMGA1. (Secondary or tertiary mammospheres do not form in control Hs578T cells, precluding analysis of these phenotypes). Next, we performed orthotopic implantations and assessed tumorigenicity with limiting dilutions. As presented above, tumors formed in both control and shHMGA1 cells when 10^7^ or 10^5^ cells were implanted. In contrast, no tumors formed in the MDA-MB-231 cells with silencing of *HMGA1* when 10^4^ cells were injected (0/3), while tumors formed in all control injections (3/3; Fig. 3C). These results indicate that silencing *HMGA1* in MDA-MB-231 cells depletes the tumor-initiator or cancer stem-like cells and further underscores the role of *HMGA1* as a key regulator of stem cell properties in aggressive, triple-negative breast cancer cells.

### HMGA1 induces a stem cell signature in triple negative breast cancer cells

**Figure 4:**
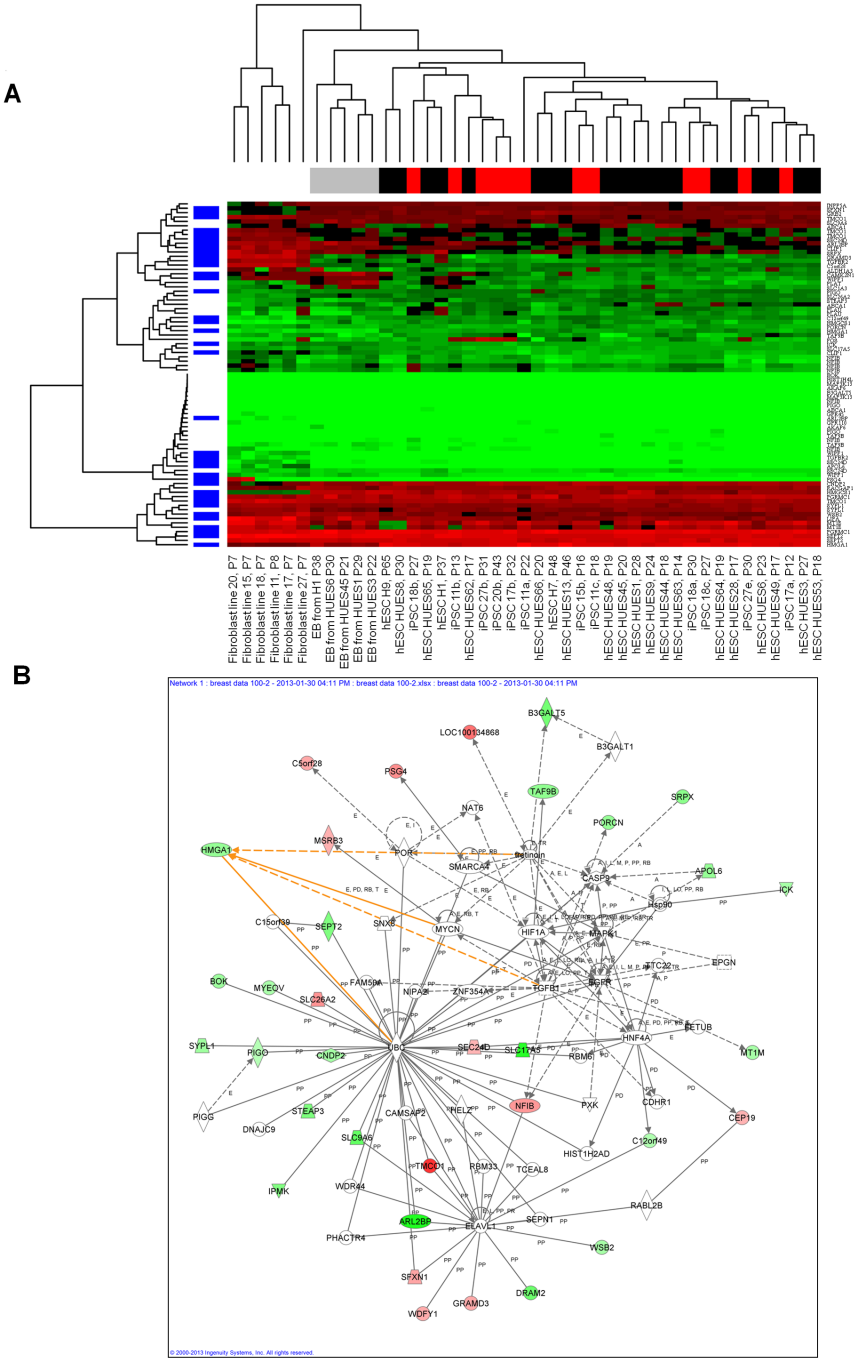
The HMGA1 signature is enriched in pluripotent stem cells, including embryonic and induced pluripotent stem cells. **A**) The HMGA signature derived from genes with the greatest expression changes in the control versus *HMGA1* knock-down cells displayed as a heat map. Green depicts down-regulation in expression, while red depicts up-regulation; black denotes little or no change in expression. The HMGA1 signature overlaps with pluripotent stem cell genes that distinguish human embryonic stem cells (hESCs) and induced pluripotent stem cells (iPSCs) from fibroblasts and embryoid bodies (EB). Genes (n = 63) were selected for the greatest changes in expression in the breast cancer cell lines with *HMGA1* knock-down as compared to the control breast cancer lines (Fig. S1). In a hierarchical clustering of fibroblasts, hESCs, iPSCs, and EBs derived from the hESCs, these genes distinguish samples by type. The majority of the HMGA1 signature genes, represented in blue along the left margin, are significantly differentially expressed between fibroblasts and human pluripotent stem cells (hESC/iPSCs; p<0.001). **B**) HMGA1 network derived from the list of differentially expressed genes using Ingenuity Pathway Analysis (IPA) with microarray gene expression data from control and *HMGA1* knock-down in MDA-MB-231 cells. From 63 differentially expressed genes as the focus gene set, the highest-scoring network was Embryonic Development, Tissue Development, and Cellular Development (score  = 69). Red nodes indicate up-regulation; green nodes indicate down-regulation. Arrows and lines denote interactions between specific genes within the network. A, activation; E, expression regulation; I, inhibition; L, proteolysis; LO, localization; M, biochemical modification; MB, membership of a group or complex; P, phosphorylation; PD, protein-DNA interaction; PP, protein-protein interaction; PR, protein-RNA interaction; RB, regulation of binding; RE, reaction; T, transcription; TR, translocation.

To globally define the transcriptional networks regulated by HMGA1, we performed gene expression profile analysis in MDA-MB-231 cells with or without *HMGA1* knock-down. To this end, we used siRNA [21], [25] and observed a rapid and significant reduction in *HMGA1* expression (Fig. S2A). *HMGA1* mRNA falls dramatically by 48 hours, with persistent decreases at 72 hours (Fig. S2A). There was also a marked decrease in HMGA1 protein at 48 and 72 hours (Fig. S2B). We therefore performed global gene expression profile analysis at 48 hours using an Affymetrix exon array (GeneChip Human Exon 1.0 ST Array) with RNA from three independent replicates of each experimental condition. To define an HMGA1 signature in breast cancer, we identified the 100 transcripts that were most differentially expressed. These 100 transcripts correspond to 63 unique genes. Because *HMGA1* is enriched in embryonic stem cells and our functional studies showed that it is required for cancer stem cell properties, we compared the HMGA1 signature of 63 genes to gene expression profiles from diverse pluripotent stem cells and differentiated cells, including embryonic stem cells (ESCs), induced pluripotent stem cells, embryoid bodies, and fibroblasts [35]. As shown, unsupervised cluster analysis of these genes separates the samples by cell type with a clear distinction between pluripotent stem cells and differentiated cells. Moreover, the HMGA1 signature is highly enriched in pluripotent/embryonic stem cells (p<0.001; Fig. 4A). We validated a subset of the HMGA1 signature genes using quantitative RT-PCR, and found differential expression similar to the microarray gene expression results in all cases (Fig. S2C). These findings suggest that HMGA1 drives tumor progression by inducing stem cell transcriptional networks.

To elucidate cellular pathways regulated by HMGA1 in breast cancer, we analyzed the HMGA1 signature with Ingenuity Pathway Analysis (IPA, Ingenuity Systems, http://www.ingenuity.com). From the top list of differentially regulated genes, 2 pathways had significant network scores (69 and 46, respectively; Fig. 4B and Fig. S3). The highest scoring network was embryonic development, tissue development, and cellular development. The top molecular and cellular functions were cell death and survival and cellular movement, while the top physiologic system development and functions included: 1. nervous system development and function, 2. organ morphology, and 3. embryonic development. In this network, the most down-regulated molecule was ARL2BP or ADP-ribosylation factor (ARF)-like 2 binding protein. This protein is a member of a functionally distinct group of RAS-related GTPases, called the ARF family. ARL2BP protein binds to ARL2.GTP with high affinity and plays a role in the nuclear translocation, retention and transcriptional activity of STAT3 [36]. Notably, we showed that HMGA1 induces *STAT3* expression in lymphoid tumorigenesis, and STAT3 inhibitors are cytotoxic to the HMGA1-driven tumor cells [24]. TMCO1 or transmembrane and coiled-coil domains 1 protein was the most up-regulated protein in this network. Although its function is not known, it is associated with breast cancer cells [8882811 GEO Profiles-NCBI]. TGFβ1 is a major node and this protein is up-regulated in diverse cancers and thought to promote invasion, migration, EMT and tumor progression [37]. EGFR and MAPK are other important nodes that are activated in cancer and mediate proliferative signals [9]. Another central node was HIF-1 alpha, a key factor involved in angiogenesis during tumor progression and vascular development during embryogenesis [38]. In addition, Myc was identified as a major node and prior studies found that not only does cMYC induce *HMGA1* expression [12], but HMGA1 also directly up-regulates *cMYC* expression [32]. Myc also has a well-defined role in breast [39]–[40] and other diverse cancers [41] as well as in embryonic stem cells [41]–[42]. Thus, our pathway analysis further confirms the important role for HMGA1 in regulating embryonic stem cell networks during tumor progression in breast cancer.

## Discussion

Here, we report for the first time that silencing *HMGA1* induces a rapid and dramatic reprogramming of highly proliferative, invasive, mesenchymal-like breast cancer cells to more differentiated, slowly growing, epithelial-like cells. We also found that knock-down of *HMGA1* has profound effects on oncogenic properties associated with both tumor initiation (orthotopic tumorigenesis) and tumor progression (migration, invasion, and metastatic progression). In fact, the *in vivo* effects on metastatic progression were even more pronounced than the effects on primary tumorigenesis, thus highlighting the role of *HMGA1* in tumor progression. The changes induced by silencing *HMGA1* are among the most striking alterations reported to date with knock-down of *HMGA1* or most other oncogenes for that matter, both in degree and rate of onset. The profound effects could be related to our efficient, viral-mediated delivery of shRNA to repress *HMGA1*. In addition, triple negative breast cancer cells may be highly dependent upon *HMGA1* and related pathways for their oncogenic properties. Indeed, a study from the Broad Institute at MIT identified HMGA1 as a key transcription factor enriched in triple negative breast cancer [3]. Moreover, expression of *HMGA1* and 8 additional genes predicted poor outcomes in breast cancer, as well as brain and bladder cancer. Prior studies using antisense or dominant-negative approaches in triple-negative breast cancer cells (MDA-MB-231 or Hs578T) also showed that anchorage-independent cell growth or colony formation are inhibited by *HMGA1* repression [15]–[16]. We also have preliminary evidence demonstrating that *HMGA1* expression correlates with more advanced nuclear grade in primary tumors (Asch & Resar, unpublished data).

Emerging evidence further indicates that HMGA1 is important in maintaining a de-differentiated, pluripotent stem-like state [32]. A recent landmark paper demonstrated that HMGA1 is required for cellular reprogramming of somatic cells to induced pluripotent stem cells (iPSCs) by the Yamanaka factors [31]. Blocking *HMGA1* expression or function prevents the derivation of iPSCs. In normal embryonic stem cells in culture and during the reprogramming process to iPSCs, HMGA1 activates expression of stem cell transcriptional networks. Recent studies also found that tumor progression and an epithelial-mesenchymal transition (EMT) involves transcriptional networks important in stem cells [3], [9]–[10], [32]–[33]. The first evidence linking HMGA1 to EMT came from an important study in 2001 in MCF-7 breast cancer cells, which demonstrated that forced expression of *HMGA1* results in metastatic progression and histologic changes consistent with EMT in the epithelial MCF-7 breast cancer cell line [16]. This group also found that HMGA1 induces changes in classes of genes involved in tumor progression. More recently, studies in colon cancer showed that HMGA1 is required for tumor progression and stem cell properties [10]. Here, we discovered that HMGA1 is required for mammosphere formation, including secondary and tertiary mammospheres in MDA-MB-231 cells. We also found that silencing HMGA1 depletes tumor initiator/cancer stem cells, indicating that targeting HMGA1 in breast cancer therapy could have an important impact on the cancer stem cell population, which is believed to be the basis for refractory disease in diverse tumors. These functional studies are corroborated by the HMGA1 signature and pathway analysis demonstrating that HMGA1 orchestrates transcriptional networks important in stem cells and metastatic progression.

There is a dire need to understand the molecular underpinnings of metastatic progression because this is the major cause of death in patients with cancer. Although cancer is a highly complex and heterogeneous disease, with significant heterogeneity even within a single tumor, increasing evidence indicates that common, central pathways exist that could serve as “Achilles heels” or rational therapeutic targets in diverse tumors. Our studies presented here underscore the fundamental role for HMGA1 in tumor progression in preclinical models for aggressive, triple negative breast cancers. This work, together with prior studies in diverse tumors, provide compelling evidence that HMGA1 is a master regulator in the evolution of primary tumors to metastatic disease. Further studies are now needed to develop approaches to target HMGA1 in cancer.

## Materials and Methods

### Ethics statement

All animal experiments were conducted in accordance with a protocol approved by the Johns Hopkins University Animal Care and Use Committee (protocol #MO11M270). Mice were housed in a sterile environment where they had free access to food and water as outlined in our institutional guidelines.

### Cell culture and proliferation assay

Cells (MDA-MB-231 and Hs578T) were cultured as recommended (ATCC). For proliferation assays, cells were seeded (7,500/well) and counted using an automated cell counter (Nexcelom). Each experiment was done in triplicate and performed at least twice.

### RNA Interference

The short-hairpin RNA interference vector for HMGA1 targets 5′-CAACTCCAGGAAGGAAACCAA-3′ and has been described elsewhere [43]. Virus was prepared as previously described. The empty vector was used as a negative control as we described [10]. Polyclonal, transduced cells were selected and maintained in puromycin (1 ug/ml). To ensure that the effects of silencing HMGA were not a result of a single clone, independent, polyclonal transductions were done at least twice for each experiment. All functional experiments were performed in duplicate or triplicate and replicated after a repeat transduction experiment and polyclonal selection of shRNA or control cells. Repression of HMGA1 was confirmed in each case at the level of gene expression (qRT-PCR) and Western analysis.

### Migration and invasion assays

Invasion assays were performed as previously described [25] with the following modifications. Briefly, 15,000 cells were resuspended in serum-free media (500 µl) and placed in the upper chamber of a 24-well BD BioCoat™ Matrigel™ Invasion Chamber coated with Matrigel. Invasion was calculated as the percentage of total cells that invaded into the bottom chamber containing complete media with serum. Migration was performed similarly, except that Matrigel was omitted.

### Orthotopic tumorigenicity and metastatic foci experiments

Cells (suspended in 75 μl phosphate-buffered saline (PBS)) were implanted into murine (NOD-scid IL2Rgamma^null^) mammary fat pads with an equal volume of Matrigel. Tumor volumes (calculated by 4/3 π× length/2× width/2× depth/2) were monitored daily until they reached 1–1.5 cm^3^, after which mice were euthanized. The presence of tumor foci within the lung was analyzed histopathologically. For tail vein injection experiments, cells (10^6^) were resuspended in PBS (150 μl). Mice were euthanized after 3 weeks, and lungs were examined histopathologically.

### Mammosphere Assay

Mammosphere assays were performed as previously described [34] and spheres (>50 µm) were counted.

### Western Analysis

Western blots were performed as previously described [10], [12], using commercial antibodies to HMGA1 (Abcam) and β-Actin (Cell Signaling), both at a 1:1000 dilution.

### Gene Expression Analysis with Quantitative, Reverse Transcription PCR

Total RNA was isolated using the Direct-zol RNA MiniPrep kit (Zymo) and analyzed by qRT-PCR as we previously described. The expression level of each gene was normalized to the human *RPLP0* (Applied Biosystems) or *β-actin* gene. Primers for *E-Cadherin*, *Snail*, and *Vimentin* were previously described [10], [33].

### HMGA1 knock-down and gene expression profile analysis

*HMGA1* was knocked-down in MDA-MB-231 cells using siRNA as we previously described [10], [21], [25]. RNA was isolated and hybridized to the Affymetrix exon array (GeneChip Human Exon 1.0 ST Array) as previously described. Expression data was pre-processed using the robust microarray (RMA) algorithm [44], as implemented in the *oligo* software package [45] available from Bioconductor [46] and annotated to the most recent human genome using the getNetAffx function in the oligo package. Microarray data were uploaded to Gene Expression Omnibus (GSE45483). Expression profiles from HMGA1 knock-down cells were compared to control cells treated with the vector RNA using Bayes modified t-tests and the *limma* package from Bioconductor [47]. We focused our analysis on the 100 most differentially expressed transcripts.

To compare this signature to genes expressed in a panel of embryonic stem cells, induced pluripotent stem cells, embryoid bodies, and fibroblasts, we downloaded expression profiles for a 43 sample study (GSE25970) [35] from the Gene Expression Omnibus [48]. Unsupervised cluster analysis was performed on all probes annotated to the 63 genes in the HMGA1 panel using agglomorative clustering with complete linkage. Euclidean distance and t-tests were used to compare probe-specific expression in stem cells and fibroblasts.

Pathway analysis of differentially expressed genes was performed using Ingenuity Pathway Analysis (IPA, Ingenuity Systems, http://www.ingenuity.com) as we described [9]. IPA scores were generated for each network and indicate the likelihood that the focus genes present in the network could occur by chance alone. A score of ≥3 is considered significant because it represents a 1/1,000 chance that the network contains specific focus genes by random chance alone [9].

## Supporting Information

Figure S1 **Silencing** ***HMGA1*** **in MDA-MB-231 blocks the formation of foci to the lung following tail vein injections. A**) Lung foci were enumerated 3 weeks following tail vein injections of control or shHMGA1 MDA-MB-231 cells (n = 3 for control mice; n = 4 for shHMGA1 mice). **B**) Graph of the mean number of tumor foci±standard deviation shows a striking decrease in foci following injection of shHMGA1 MDA-MB-231 cells as compared to controls (p = 0.007).
(TIF)

Figure S2 **Silencing** ***HMGA1*** **in MDA-MB-231 results in significant repression in** ***HMGA1*** **mRNA and protein, with alterations in gene expression. A**) Independent replicate experiments of MDA-MB-231 cells with or without *HMGA1* knock-down result in silencing *HMGA1* at the level of mRNA. **B**) HMGA1 protein is also repressed following treatment with siRNA. **C**) Validation of genes in the HMGA1 signature shows that gene expression assessed by quantitative RT-PCR (qRT-PCR) parallels that of the microarray results. **D**) Table comparing differential expression of the HMGA1 signature identified by microarray and qRT-PCR.
(TIF)

Figure S3 **HMGA1 network derived from differentially expressed genes in MDA-MB-231 with or without** ***HMGA1*** **knock-down.** From 63 differentially expressed genes as the focus gene set, the second highest-scoring network was Cardiovascular Disease, Cell Death and Survival, and Nervous System Development and Function (score  = 46). Colors, arrows, lines and abbreviations are described under Figure 4B. NF-κB, ERK, and MAPK are major nodes, which have been identified in prior studies of global gene expression profiles mediated by HMGA1 [9].
(TIF)
