# Supplementary figures and images for: HMGA1: A Master Regulator of Tumor Progression in Triple-Negative Breast Cancer Cells
Source: PLoS One. 2013 May 2;8(5):e63419. doi: 10.1371/journal.pone.0063419 (PMC3642138; doi:10.1371/journal.pone.0063419)

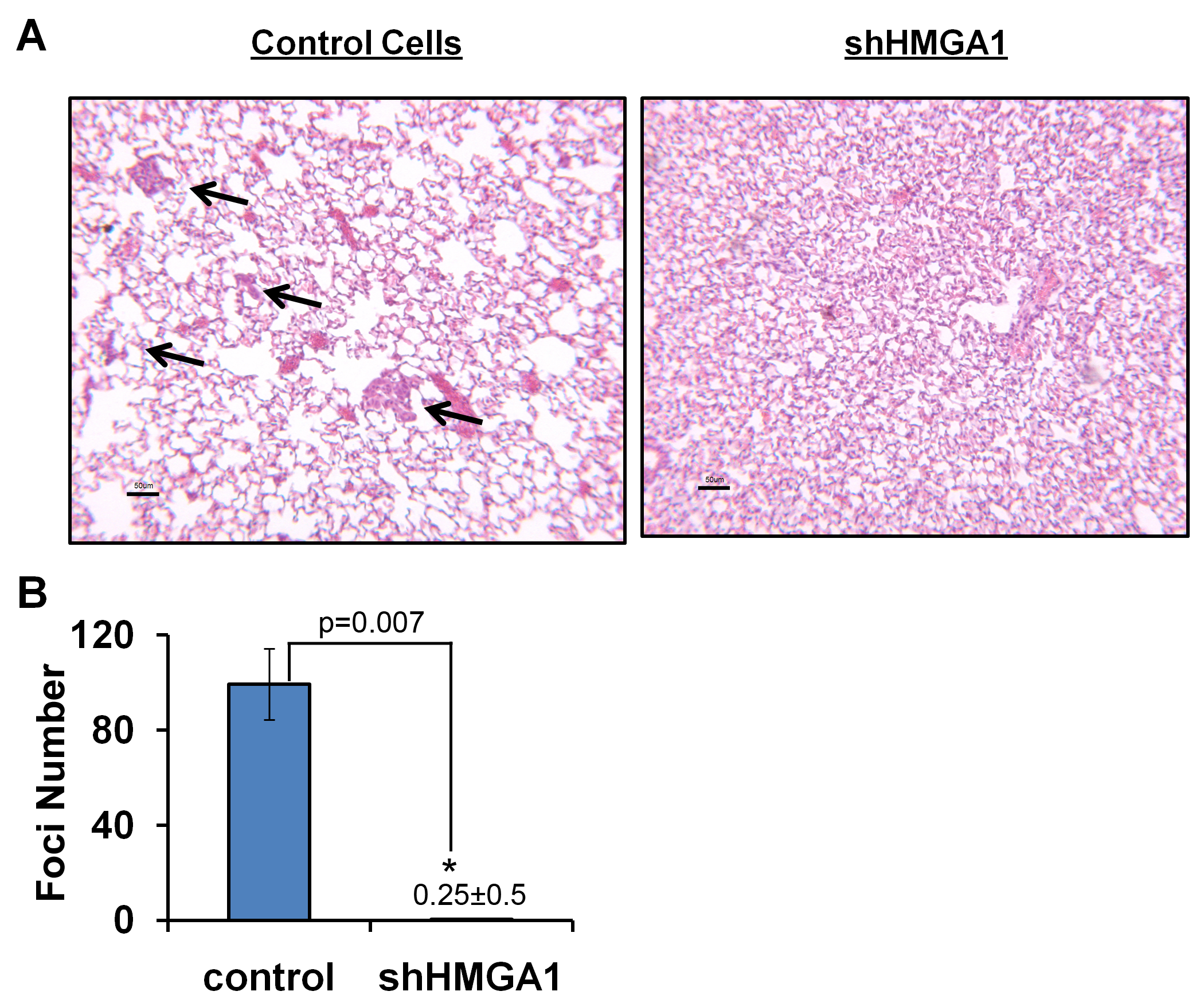

Supplement: Figure S1 — Silencing HMGA1 in MDA-MB-231 blocks the formation of foci to the lung following tail vein injections. A) Lung foci were enumerated 3 weeks following tail vein injections of control or shHMGA1 MDA-MB-231 cells (n = 3 for control mice; n = 4 for shHMGA1 mice). B) Graph of the mean number of tumor foci±standard deviation shows a striking decrease in foci following injection of shHMGA1 MDA-MB-231 cells as compared to controls (p = 0.007). (TIF) [file pone.0063419.s001.tif]

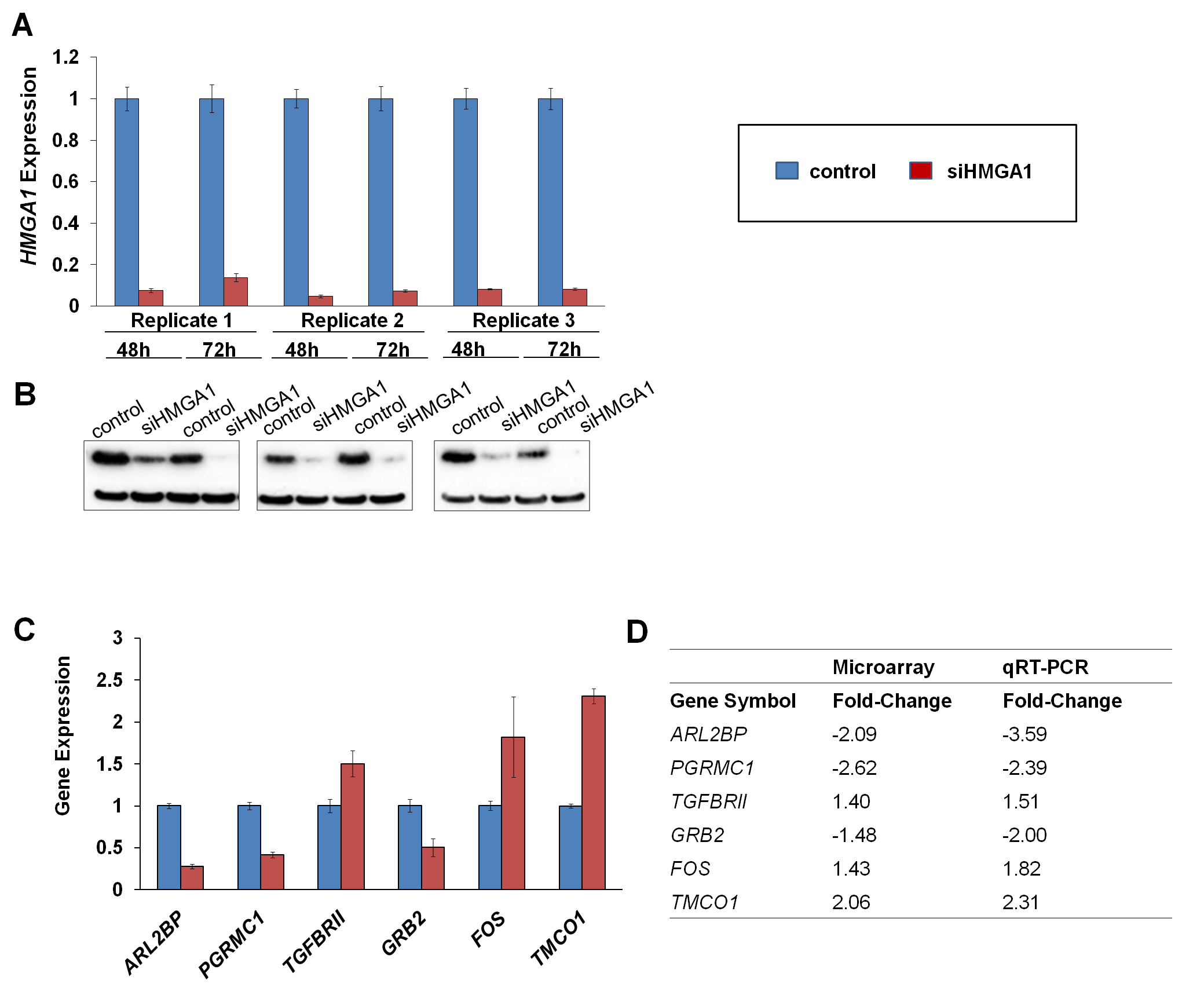

Supplement: Figure S2 — Silencing HMGA1 in MDA-MB-231 results in significant repression in HMGA1 mRNA and protein, with alterations in gene expression. A) Independent replicate experiments of MDA-MB-231 cells with or without HMGA1 knock-down result in silencing HMGA1 at the level of mRNA. B) HMGA1 protein is also repressed following treatment with siRNA. C) Validation of genes in the HMGA1 signature shows that gene expression assessed by quantitative RT-PCR (qRT-PCR) parallels that of the microarray results. D) Table comparing differential expression of the HMGA1 signature identified by microarray and qRT-PCR. (TIF) [file pone.0063419.s002.tif]

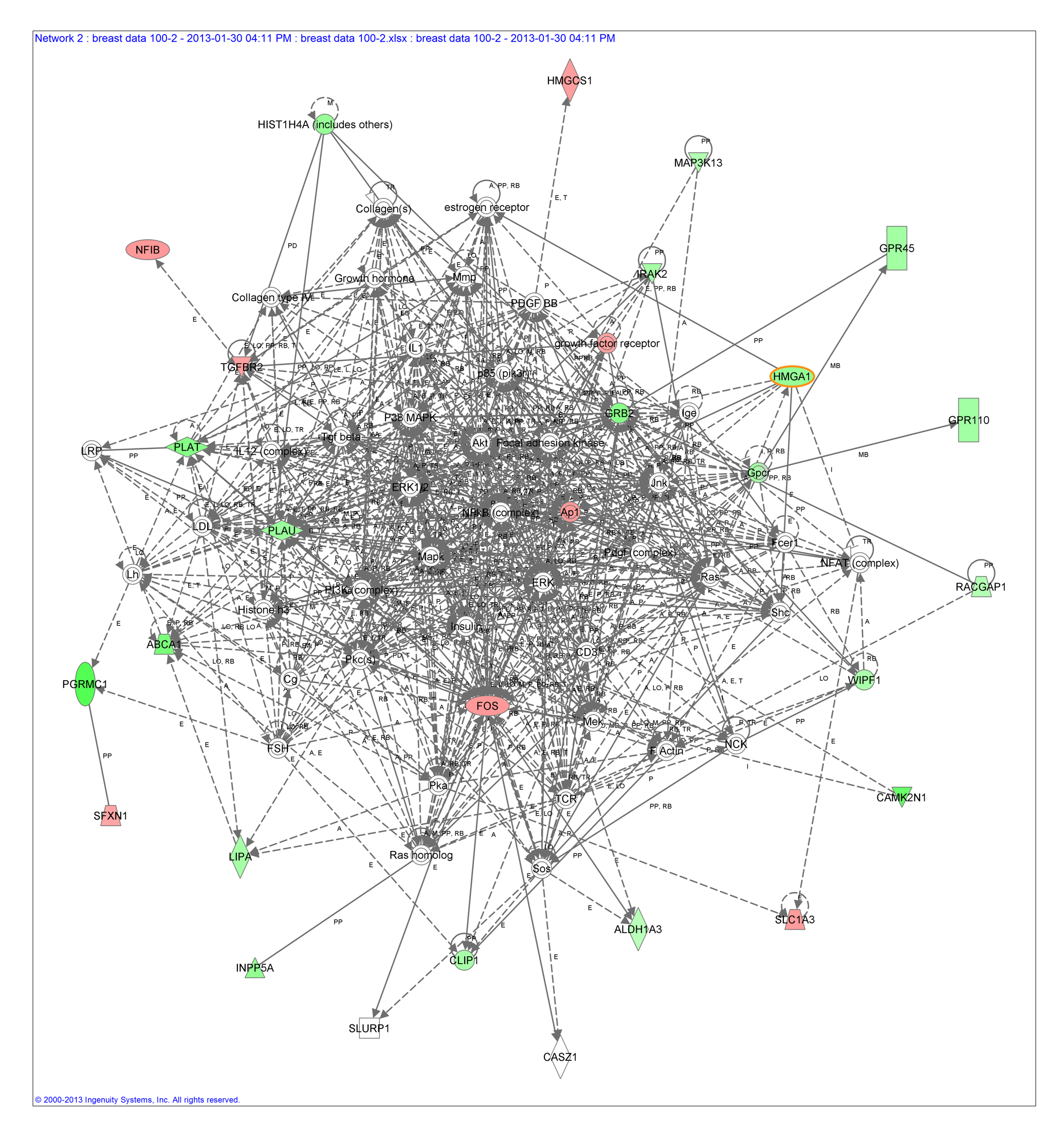

Supplement: Figure S3 — HMGA1 network derived from differentially expressed genes in MDA-MB-231 with or without HMGA1 knock-down. From 63 differentially expressed genes as the focus gene set, the second highest-scoring network was Cardiovascular Disease, Cell Death and Survival, and Nervous System Development and Function (score = 46). Colors, arrows, lines and abbreviations are described under Figure 4B. NF-κB, ERK, and MAPK are major nodes, which have been identified in prior studies of global gene expression profiles mediated by HMGA1 [9]. (TIF) [file pone.0063419.s003.tif]
